# Supplementary material for: Screening of Nutritionally Important Components in Standard and Ancient Cereals
Source: Foods. 2024 Dec 19;13(24):4116. doi: 10.3390/foods13244116 (PMC11675112; doi:10.3390/foods13244116)
Supplement: Supplementary file 1 [file foods-13-04116-s001.zip › foods-3323926-supplementary.pdf]

Supplementary Table S1. Genotypes used in experiment.

Shortcut	Genotype/Variety	Species	Short description
Dur1	Cosmostar	Durum wheat (*Triticum durum* Desf.)	ZP variety for pasta production
Dur2	NS Dur		Standard for testing
Dur3	Agaton		ZP variety, high tolerance to low temperature
BW1	Zemunska rosa	Bread wheat (*Triticum aestivum* L.)	ZP variety, tolerant to drought
BW2	NS40s		Standard for testing
Trit1	Admiral	Triticale (× *Triticosecale* Wittm. ex A. Camus.)	ZP variety, early maturity, drought tolerant
Trit2	Agrounija		Medium early variety, tolerant to low temperature.
Trit3	Odisej		Standard for testing
Bar1	Apolon	Hullessbarley (*Hordeum vulgare* L.)	ZP variety, medium early, tolerant to lodging
Bar2	Osvit		Medium early variety, tolerant to lodging
Bar3	35/Ig		ZP line in breeding process
Bar4	Nektar	Hulledbarley (*Hordeum vulgare* L.)	ZP variety, medium early, for beer production
Em1	Emmer FON	Emmer wheat (*Triticum dicoccum* Schrank ex Schübl.)	Line, Greek origin
Em2	Emmer LP2-1-5		Variety, Montenegro Gene-bank
Spelt	Cimmyt spelt 1	Spelt wheat (*Triticum spelta* L.)	Line, Cimmyt Gene-bank
Rye1	BLR 5-15	Rye (*Secale cereale* L.)	Line, ZP Gene-bank
Rye2	BLR 14-15		
Oats1	Sopot – black oats	Oats (*Avena sativa* L.)	Variety, ZP Gene-bank
Oats2	Caramel oats		
Oats3	Brown oats		

Supplementary Table S2. Ratio between phytic acid (Phy) and essential elements in examined standard and ancient grains.

Genotypes	Phy/Ca	Phy/Mg	Phy/Fe	Phy/Mn	Phy/Zn
Dur1	2.61 ± 0.02*	0.49 ± 0.01	27.93 ± 0.16	46.68 ± 0.02	44.20 ± 0.01
Dur2	2.02 ± 0.01	0.50 ± 0.01	30.46 ± 0.14	46.67 ± 0.02	49.51 ± 0.01
Dur3	2.57 ± 0.02	0.54 ± 0.01	33.30 ± 0.22	58.73 ± 0.10	47.53 ± 0.04
BW1	1.97 ± 0.01	0.54 ± 0.01	34.86 ± 0.05	40.67 ± 0.07	48.68 ± 0.03
BW2	2.14 ± 0.01	0.49 ± 0.01	35.66 ± 0.05	41.17 ± 0.12	55.43 ± 0.06
Trit1	3.22 ± 0.02	0.55 ± 0.01	41.31 ± 0.04	46.58 ± 0.06	54.06 ± 0.03
Trit2	2.28 ± 0.02	0.49 ± 0.02	59.75 ± 0.06	52.53 ± 0.14	68.92 ± 0.06
Trit3	2.18 ± 0.01	0.53 ± 0.02	49.55 ± 0.05	43.13 ± 0.03	47.13 ± 0.02
Bar1	2.40 ± 0.02	0.50 ± 0.01	24.06 ± 0.63	98.38 ± 0.15	81.27 ± 0.07
Bar2	1.95 ± 0.01	0.54 ± 0.01	38.77 ± 0.50	97.75 ± 0.40	50.78 ± 0.19
Bar3	2.23 ± 0.01	0.54 ± 0.02	57.89 ± 0.61	129.89 ± 0.35	88.43 ± 0.16
Bar4	1.65 ± 0.01	0.47 ± 0.01	27.98 ± 0.43	78.74 ± 0.21	54.15 ± 0.10
Em1	2.65 ± 0.02	0.49 ± 0.01	22.82 ± 0.14	39.16 ± 0.05	45.53 ± 0.03
Em2	3.97 ± 0.03	0.60 ± 0.01	28.69 ± 0.12	43.30 ± 0.01	44.06 ± 0.00
Spelt	2.09 ± 0.01	0.44 ± 0.01	27.29 ± 0.10	39.38 ± 0.11	26.15 ± 0.05
Rye1	2.15 ± 0.01	0.52 ± 0.01	19.78 ± 0.27	51.70 ± 0.05	46.31 ± 0.02
Rye2	1.88 ± 0.01	0.51 ± 0.01	26.66 ± 0.26	56.75 ± 0.11	43.67 ± 0.05
Oats1	1.07 ± 0.01	0.46 ± 0.01	35.90 ± 0.00	35.75 ± 0.05	41.54 ± 0.02
Oats2	1.13 ± 0.01	0.46 ± 0.01	27.96 ± 0.14	44.05 ± 0.10	55.29 ± 0.04
Oats3	1.03 ± 0.01	0.42 ± 0.01	17.22 ± 0.11	30.48 ± 0.07	38.30 ± 0.03

*Average ± standard error.

Supplementary Table S3. Correlation coefficients between PC axes and tested parameters (protein, inorganic phosphorus (Pi), phytic phosphorus (Pphy), yellow pigment (YP), total phenolics (TPC), total glutathione (GSH) β-glucan (βGlu), arabinoxylan (Arab), naringin (NA),p-Coumaric acid (p-CoumA), ferulic acid (FA), quercetin (QUE)).

Tested parameter	PC1 (34.1%)	PC2 (18.9%)	PC3 (12.6%)	PC4 (10.7%)
Prot	-0.10	-0.17	0.22	-0.14
Pi	0.14	0.13	0.69	0.14
Pphy	-0.70	0.24	0.11	-0.25
TPC	0.24	0.26	0.11	0.14
GSH	0.27	0.30	0.74	0.15
DPPH	-0.14	0.25	-0.07	0.16
YP	0.28	0.19	0.28	0.48
βGlu	0.27	-0.12	0.76	0.34
Arab	0.93	-0.30	-0.30	-0.41
P	-0.19	0.27	0.85	-0.32
K	-0.21	0.18	0.14	-0.84
Ca	0.74	0.17	-0.31	-0.20
Mg	0.16	0.29	0.18	-0.72
Na	-0.12	-0.26	-0.12	0.13
Fe	-0.07	0.25	0.48	0.74
Mn	-0.06	0.33	-0.78	-0.35
Zn	-0.14	0.76	0.39	-0.22
Cu	-0.17	0.25	-0.12	0.75
NA	0.28	0.24	-0.97	0.13
p-CoumA	0.22	0.70	-0.15	0.22
FA	0.81	0.37	-0.08	-0.07
QUE	0.76	0.51	0.15	0.45
